# VCSim3: a VR simulator for cardiovascular interventions

**DOI:** 10.1007/s11548-017-1679-1

**Published:** 2017-10-27

**Authors:** Przemyslaw Korzeniowski, Ruth J. White, Fernando Bello

**Affiliations:** 1 Simulation and Modelling in Medicine and Surgery, Centre for Engagement and Simulation Science, Imperial College London, London, UK; 2 Chelsea and Westminster Hospital, 369 Fulham Road, London, SW10 9NH UK

**Keywords:** Catheter, Guidewire, Cosserat rod, VR simulator, Coronary interventions, Medical training

## Abstract

**Purpose:**

Effective and safe performance of cardiovascular interventions requires excellent catheter/guidewire manipulation skills. These skills are currently mainly gained through an apprenticeship on real patients, which may not be safe or cost-effective. Computer simulation offers an alternative for core skills training. However, replicating the physical behaviour of real instruments navigated through blood vessels is a challenging task.

**Methods:**

We have developed VCSim3—a virtual reality simulator for cardiovascular interventions. The simulator leverages an inextensible Cosserat rod to model virtual catheters and guidewires. Their mechanical properties were optimized with respect to their real counterparts scanned in a silicone phantom using X-ray CT imaging. The instruments are manipulated via a VSP haptic device. Supporting solutions such as fluoroscopic visualization, contrast flow propagation, cardiac motion, balloon inflation, and stent deployment, enable performing a complete angioplasty procedure.

**Results:**

We present detailed results of simulation accuracy of the virtual instruments, along with their computational performance. In addition, the results of a preliminary face and content validation study conveyed on a group of 17 interventional radiologists are given.

**Conclusions:**

VR simulation of cardiovascular procedure can contribute to surgical training and improve the educational experience without putting patients at risk, raising ethical issues or requiring expensive animal or cadaver facilities. VCSim3 is still a prototype, yet the initial results indicate that it provides promising foundations for further development.

**Electronic supplementary material:**

The online version of this article (10.1007/s11548-017-1679-1) contains supplementary material, which is available to authorized users.

## Introduction

### Cardiovascular interventions

Cardiovascular diseases (CVD) are the main cause of death in the developed world [1]. Minimally invasive endovascular procedures, widely adopted in diagnosis and treatment of CVDs, improve recovery time and reduce patient trauma and healthcare costs. During such procedures, endovascular clinicians insert long, thin, flexible surgical instruments—catheters and guidewires, into the patient’s vascular system. Guided by medical imaging, they then navigate the catheter/guidewire pair into the coronary arteries to treat the pathology. An effective and safe performance of these procedures requires excellent instrument manipulation skills, which are still mainly gained through an apprenticeship on real patients. Drawbacks of the apprenticeship model include high costs, reduced training opportunities, and patient safety [2]. One possible alternative is training on computer-based, virtual reality (VR) simulators [3]. The last decade has seen growing interest in the benefits of using VR medical simulators in a range of specialties, including endovascular interventions [4].

Commercial VR vascular simulators such as VIST (www.mentice.com) or AngioMentor (www.simbionix.com) have demonstrated a degree of face and content validity [5], but the ultimate realism is yet to be achieved, in particular, with respect to replicating and detecting fine motor actions, and related subtle visual and haptics cues [6]. The fundamental part of such simulators is the underlying mathematical model of the one-dimensional deformable bodies (elastic rods [7]) responsible for the behaviour of virtual catheters and guidewires. Elastic rods are characterized by having large nonlinear deformations, even if the local strains are small. This characteristic, as well as consideration of material twist and the fact that many rods practically do not stretch, makes the dynamic simulation of elastic rods challenging in real-time.

### Previous work

Physically based approaches to real-time elastic rods range from mass-spring models [8–10], rigid multi-body serial chains [11, 12], spline-based formulations [13], to Cosserat theory based models [14–19]. More recently, solutions integrating the Cosserat theory with Position-Based Dynamics [20] were presented by Umetani et al. in [21] and by Kugelstadt and Schomer [22].

In the field of endovascular simulation, Dawson et al. [23] proposed a catheter model based on a multi-body system composed of rigid bodies and joints, which requires many links in order recreate a high degree of flexibility. Wang et al. [24] and Luboz et al. [25] showed the possibility of simulating an elastic rod in real time and with visually correct accuracy using a mass-spring model. Wang et al. [24] recreated the material twist in MSS using a scalar torsion angle parameter.

Nowinski and Chui [26] applied a linear elasticity finite element representation, which assumes that the instruments move only with small displacements. Duriez et al. [27] introduced a static nonlinear deformable beam model resulting in an accurate simulation of bending and twist deformations, while Lenoir et al. [28] applied this approach to simulate interactions between catheter and guidewire by modulating material properties of the finite element method (FEM) model. Alderliesten et al. [29, 30] simulated rods, including friction, as a set of straight, non-bendable, incompressible beams with perfect torque control using a quasi-static approach. Later, Li et al. [31] improved this approach by using a FEM-based numerical solver. More recently, Duratti et al. [32] applied a solution closely resembling the CoRde model [17] to real-time interventional radiology simulation and [33, 34] adapted the approach in [18] to simulate catheters and guidewires.

Before a simulator can be integrated into an educational program, it is recommended that its validity be determined. A study by Schout et al. [35] provides a critical review of the literature and the main experiences relating to the validation of simulators. The study states that there is general agreement in the literature that a distinction can be made between subjective and objective approaches to validation. The subjective approaches examine study participants opinions, while objective approaches are used in prospective experimental studies. In the subjective case, after performing a procedure on a simulator, the study participants are asked to complete a questionnaire about their experience with the simulator. The questionnaire judges the degree of resemblance between the simulated and the real surgical procedure (face validity) and examines the level to which the simulator covers the subject matter (content validity) [36]. The study states that the literature does not offer any general guidelines concerning methods, settings, and data interpretation regarding these validation methods.

The objective approaches involve an experiment to determine whether the simulator can discriminate between different experience levels (i.e. between expert vs. novice participants, construct validity) or, ultimately, to assess the outcomes of simulator training by measuring performance, e.g. on a real patient (transfer validity). The study concludes that objective validation studies were mainly characterized by a large variety of methods and parameters used to assess validity, and in the definition and identification of expert and novice levels of performance. Moreover, [35] lists only few cases of transfer validity studies due to ethical and legal issues restricting these types of studies.

**Fig. 1:**
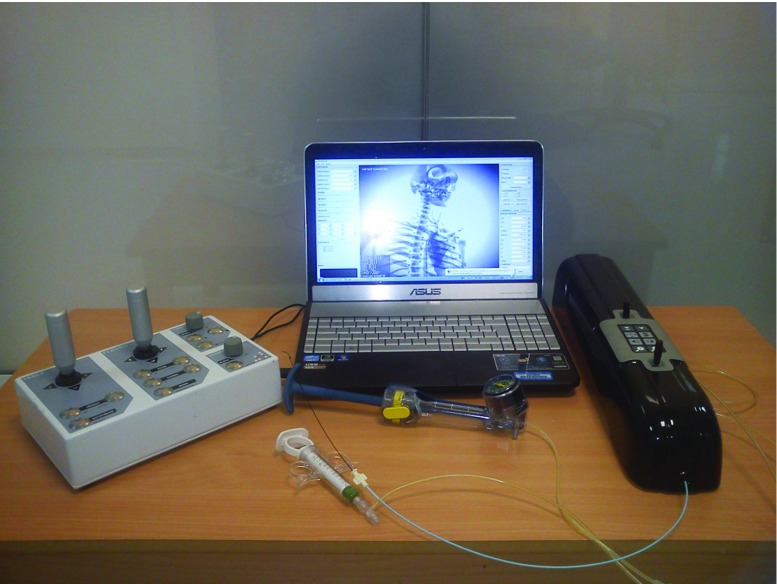
VCSim3 complete set-up including the simulator software running on the laptop, VSP haptic device, fluoroscopic view console, balloon inflation device, and contrast injection syringe

In this paper, we describe our VCSim3 simulator, including its overall design, haptic interface, virtual instruments model, as well as implementation details. Next, we present results of the underlying model behaviour along with its computational performance. This is followed by the application of the VCSim3 to a complete angioplasty procedure and details of initial validation results.

## Methods

### Simulator overview

The VCSim3 set-up (Fig. 1) consists of real-time simulation software, fluoroscopic view console and a VSP haptic device (*Vascular Simulation Platform*, www.mentice.com). The cross-platform software is written in Java, with performance critical sections implemented in C/C++, and can exceed haptic interactive rates (0.5–1 kHz) on a mid-range PC. It is responsible for X-ray visualization and simulation of the virtual catheter/guidewire pair, interactions between instruments and vessels walls, contrast medium propagation, balloon inflation, and stent deployment.

**Fig. 2:**
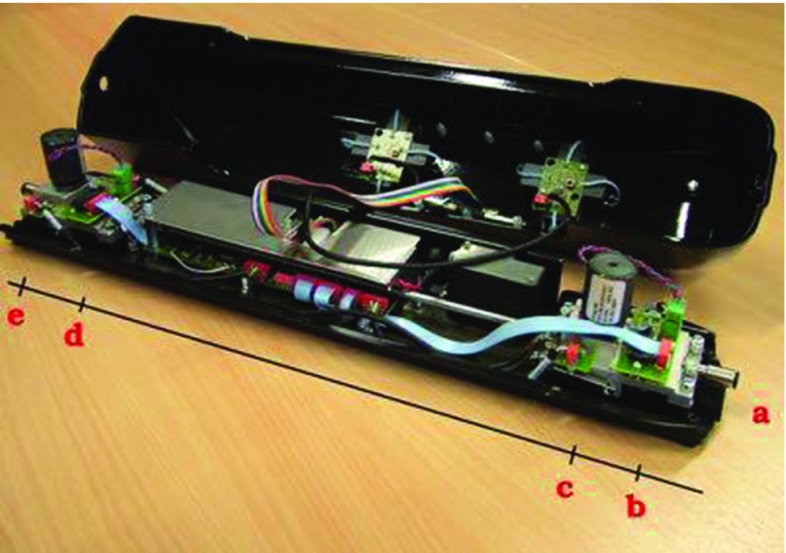
The VSP with removed chassis. Photography by Mr. Hafiz Harun

The operator controls the virtual catheters and guidewires using the VSP haptic device connected through a USB port. The VSP can track real endovascular instruments; however, it needs to be calibrated for particular diameters prior to use. The instruments can be pushed, pulled and rotated. Inside (Fig. 2), the VSP is fitted with two optical sensors for instrument tracking and two force feedback mechanisms. The first sensor (b), placed near to the insertion slot (a), tracks the catheter. The second one (d), located approximately 30  cm further, tracks the guidewire. This results in a non-constrained guidewire tracking length, but limits the effective catheter tracking to 30  cm. The force feedback is generated by the motors (c, e) simply clamping the instruments. The VSP is equipped with a pressure sensor, to detect balloon inflation and a flow sensor to identify use of the syringe for contrast injection.

**Fig. 3:**
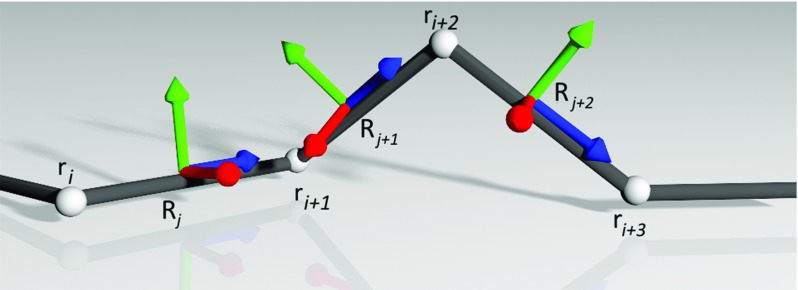
Material frames adapted to the Cosserat rod centreline

An optional fluoroscopic view console (Fig. 1) adapted from the VIST simulator (www.mentice.com) is equipped with two joysticks allowing for panning and rotating the X-ray view, as well as a set of buttons for controlling zooming and gamma. The view manipulations may also be done using the mouse and keyboard.

### Virtual catheter and guidewire modelling

We chose to model our virtual catheter and guidewire based on Cosserat Theory—a solid theoretical foundation considered as a final step in the formulation of a modern theory of elastic rods [37]. In brief, the Cosserat theory of elastic rods assumes that each point lying on a rod centreline has its own local coordinate system—the material frame—which is moving together with this point when the rod is undergoing a deformation. By comparing the frames, the bend and twist deformations of the rod can be quantified. For discrete computer implementation of the Cosserat theory, we chose an adapted version of the CoRdE—a fast, dynamic and elegant computer model proposed in [17], which, in our case, assumes inextensibility. This model was previously applied by our group to simulate a flexible endoscope in natural orifice transluminal surgery with good results [38, 39].

CoRdE represents the centreline of the rod as a series of mass points $\mathbf{r}_i \in {\mathbb {R}}^{3}$ and material frames associated with the edges between two adjacent mass points $\mathbf{R}_j \in {\mathbb {R}}^{3\mathbf{x}3}$. The material frames are governed by a quaternion, as shown in Fig. 3. The mass points are only loosely coupled with the quaternions and need to be explicitly realigned to each other. In CoRdE, this is realized by the parallel constraint imposed by the penalty method which, yielding an additional energy term, generates restitution forces and torques that accelerate the centreline and material frames to the equilibrium. Some authors argue that such an explicit representation of the centreline simplifies the overall implementation, internal friction calculations, and rods visualization. Additionally, it facilitates the handling of contacts and looping phenomena [17, 18]. Please refer to the original paper [17] for further details on the CoRdE implementation.

#### Modified inextensible CoRdE

The original CoRdE model is extensible and compressible, which is not desirable for the simulation of catheters or guidewires. In this model, the reduction in stretch or compressibility and, due to the aforementioned penalty method, the simulation of stiff or inextensible rods would introduce an additional stiffness to the system. As a result, the stable simulation would require a smaller time step what would degrade the performance.

To address this, we modified the original model to make it inextensible and incompressible like real endovascular instruments. We used a block iterative Projected Gauss–Seidel method (PGS [40, 41]) to solve the resulting system of constraints and collisions. The PGS method is popular for computing constraint and contact forces in interactive real-time rigid body simulation software, since it is very efficient, both computationally and memory wise, as well as robust, being able to deal with bad or erroneous problems [42]. Moreover, an iterative approach enables linearizing and solving the nonlinear constraints multiple times within one time step to improve simulation accuracy.

The penalty method, governing rods stretch, was replaced by a chain of distance constraints. For accuracy and performance, similarly to [43], the constraints were arranged in a tridiagonal banded system of equations. As this system contains only equality constraints, it is efficiently solved in a global manner (i.e. all distance constraints at once) as a first step of each solver iteration by a direct linear algebra library (LAPACK, www.netlib.org).

**Fig. 4:**
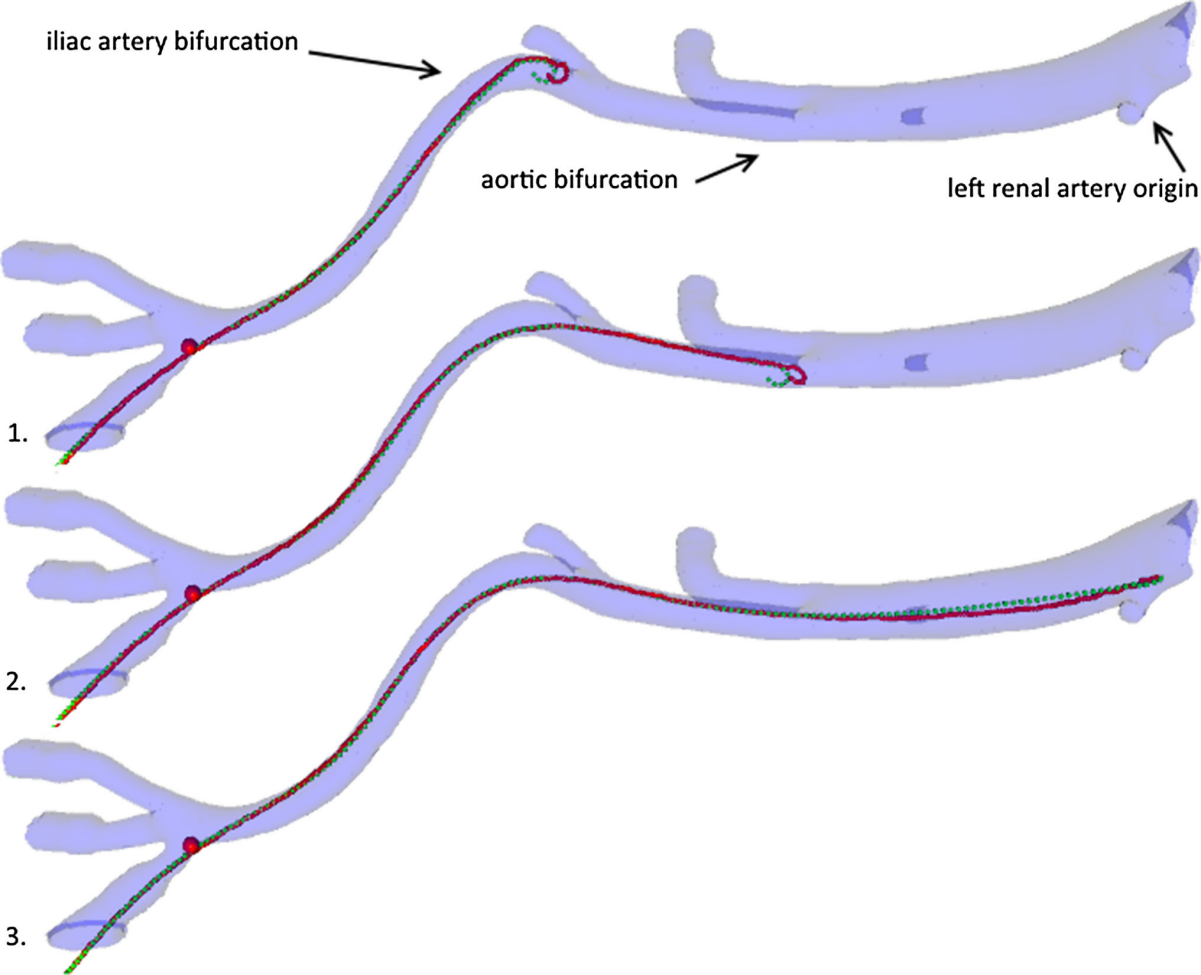
Reconstructed 3D geometry of the phantom showing the centreline of the guidewire in red and the simulated instrument centreline in green

After exactly satisfying all the distance constraints, the concentric constraints are applied. Their task is to keep the guidewire inside the catheter allowing only for coaxial sliding movement. The concentric constraints, which can be also thought of as zero-length distance constraints, generate velocity changes (impulses) perpendicular to the instruments centrelines between all the mass points of the guidewire inside the catheter, and the two nearest corresponding points on the catheter. These two points receive a fraction of the impulse proportional to the distance from the guidewire point.

Finally, the collisions with vessels constraints including Coulombian friction approximation are applied locally [40]. Collision detection between the instrument and the vessels is based on a bounding volume hierarchy (BVH) of axis-aligned bounding boxes (AABB) as suggested in [44], which guide the broad-phase collision detection stage (sphere vs. AABB). For rapidly moving coronaries, a dynamic ‘fat’ AABB tree was implemented. For performance reasons, the tree structure is pre-calculated and optimized during the simulation start-up and remains unchanged throughout the procedure. Only the positions and volumes of bounding boxes are recomputed during each step to match the shape of moving coronaries. In a narrow phase, a standard sphere vs. triangle checks are performed. The collision response calculates the weighted average of normal vectors of all colliding triangles. The resulting collision vector is then used by the constraints solver to prevent the mass points of the virtual instruments from moving along this direction.

**Fig. 5:**
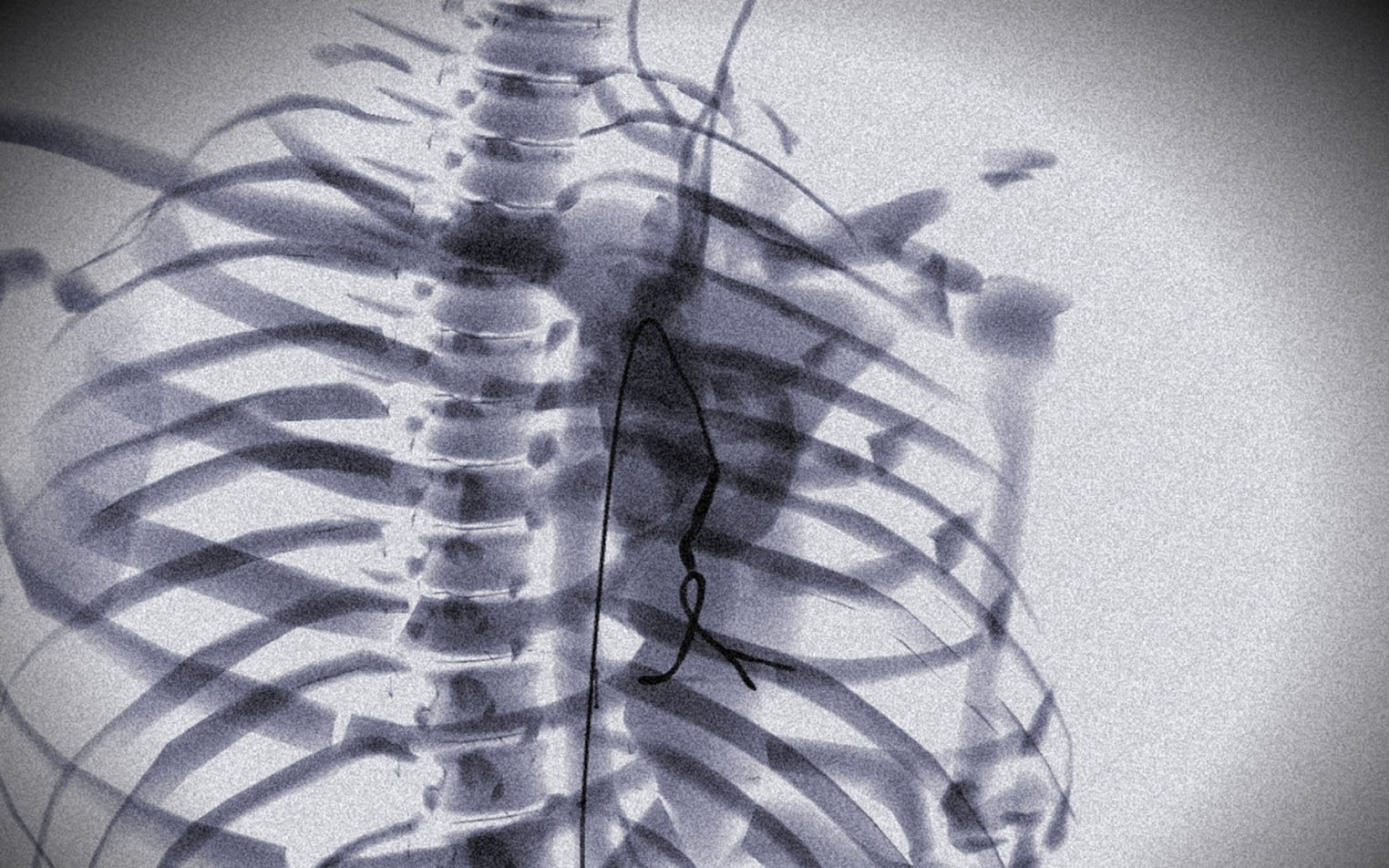
The simulated fluoroscopy screen of VCSim3 showing a catheter inserted into the RCA with contrast injected

In our opinion, this approach offers a good compromise between computational efficiency [17] and simulation accuracy [45]. We effectively ensure rod inextensibility and incompressibility, but since in our application there is no need for stiff rods, the computationally efficient penalty method, governing parallel constraints, was left intact. Moreover, the penalty method can be applied locally, which might be beneficial for potential massively parallel implementation of this model. A possible disadvantage is the need for an additional numerical spring constant parameter $$K_p $$ influencing the rod behaviour. In [45], the authors eliminate the penalty method by using a more accurate, but less efficient and global (i.e. harder to parallelize) Lagrange multipliers approach enabling the simulation of stiffer rods and larger time step. We tackle this issue by means of parameter optimization.

#### Parameter optimization

The behaviour of nine commonly used instruments were recreated by optimizing their mechanical parameters with respect to their real counterparts scanned in a vascular silicone phantom model using X-ray CT imaging [46], as shown in Fig. 4. The average RMS distance between simulated and reference centrelines was minimized with respect to the material property parameters of the model. As different parameters can be applied to a number of the end mass points of the rod, the optimization considered softer tips of real instruments, which reduce perforations of vessel walls. It also accounted for the performance requirements for real-time usability and stability. Please refer to [46] for the details, which are briefly summarized below.

The optimization consisted of the minimization of an error function calculated as the root-mean-square (RMS) distance between the simulated mass points and nearest points on the reference centreline, dependent on the following model parameters [17] Young modulus ($E_b$), radius (*r*), density (*d*), spring constant of the parallel constraint ($K_{p}$) and the ratio between the Young modulus of the tip and the shaft ($\alpha$), which modelled the floppy nature of the tips of the instruments. As both catheters and guidewires exhibit high resistance to twisting, the shear modulus (*G*) was not considered in the optimization. Instead, it was set to highest possible numerically stable value.

The error function was an average RMS distance between simulated and reference centrelines:

1 $$\begin{aligned} \hbox {f}\left( {E_b ,r,d,K_p ,\alpha } \right) =\sqrt{\frac{1}{N}{\sum \nolimits _\mathrm{i=1}^{N}} \left( {\hbox {minDist}\left( {\hbox {p}_\mathrm{i}^\mathrm{s} } \right) } \right) ^{2}} \end{aligned}$$

where *N* is the number of simulated mass points inserted into the phantom and the function $\hbox {minDist}\left( {\hbox {p}_\mathrm{i}^\mathrm{s} } \right)$ returns the nearest Euclidean distance to the reference centreline from the position of the *i*-th simulated mass point—$\hbox {p}_\mathrm{i}^\mathrm{s}$.

### Supporting solutions

For the simulator to be a fully immersive training tool, in addition to a realistic instrument model and haptic interface, it needs to provide various features that enable the simulation of a full procedure. The visualization was created with several layers, allowing the trainee to select how much detail they wanted to see. Off-the-shelf models of the skeleton and an animated heart were utilized, together with a custom X-ray shader to create a simulated fluoroscopy image, as shown in Fig. 5.

Several attempts were made to segment a detailed, animated heart mesh including the coronary arteries from a combination of CT and MRI images using computer vision methods. However, this approach did not yield the necessary results in the available time. Therefore, a hybrid approach was used instead. A static, coronary arteries mesh was segmented from a real patient data set and manually, frame by frame, overlaid onto the surface of an off-the-shelf animated (24 frames) heart model (www.3dscience.com) using a 3D software package (3ds Max, www.autodesk.com).

**Fig. 6:**
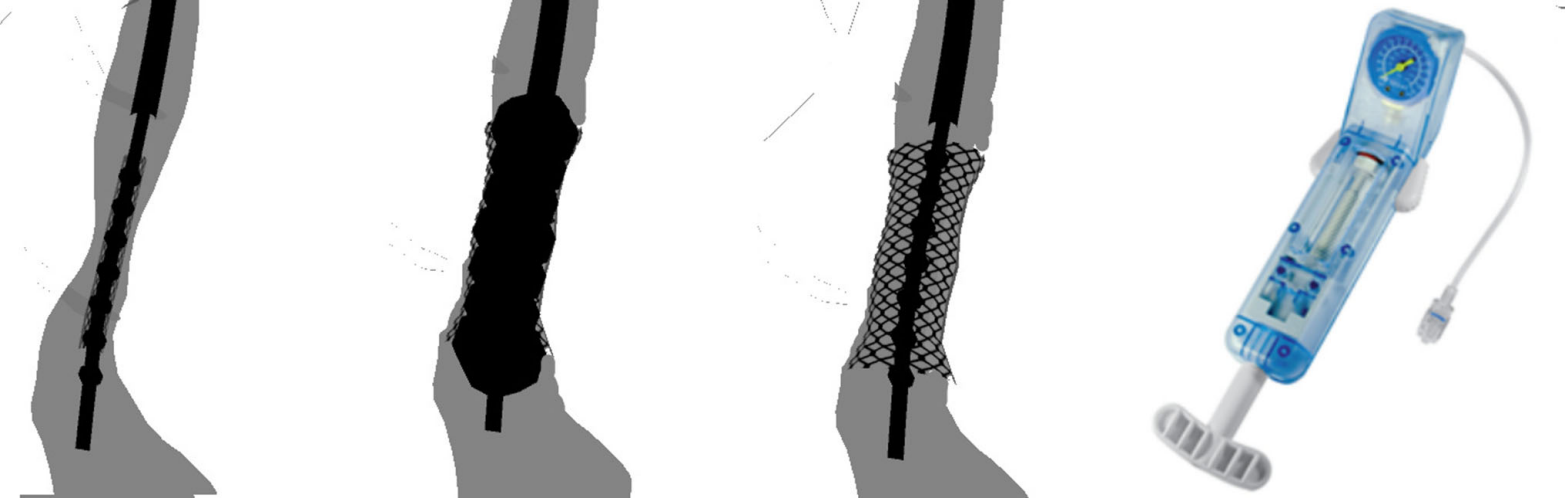
Three stages of stent deployment: positioning the balloon with stent in a stenosis (left), inflating the balloon (middle), deflating the balloon and releasing the stent (right)

The propagation of the contrast is implemented using a model based on [47]. An ordered set of spheres are fitted into the coronary arteries and each sphere is described by its index, position, radius and pre-calculated flow speed. The opacity of each sphere is varied according to its volume and the amount of contrast currently flowing through it. The speed of this propagation is dependent on the current heartbeat phase.

To enable a full angioplasty simulation, a balloon and stent must also be modelled. The balloon is modelled as a series of spheres attached to the distal end of the guidewire. During inflation, the spheres are gradually increased in size and dynamically adjusted to fit into the shape of the vessels, using iterative collision detection. At the same time, the mesh model of a stenosed vessel is gradually interpolated into the healthier one. The stent is also modelled using spheres and follows the balloon until release, when its mass points are separated. A mass-spring model is implemented to ensure it remains in place and follows the movement of the vessels. Figure 6 shows the main stages of stent deployment.

## Results

### Virtual catheter and guidewire performance

Our modified CoRdE implementation enables real-time simulation at haptic interactive rates. Qualitative observation of the simulation shows a nearly immediate response to user manipulations at the proximal end, efficient and unified bending, twisting and collision handling, and easy parameterisation of the mechanical properties of the rod to recreate the behaviour of real instruments. Below we provide quantitative results pertaining to the positional accuracy of the virtual instrument and the computational performance of the model.

Results show the parameter-optimized virtual instruments exhibit near-sub-millimetre positional accuracy, with errors likely to be caused by the accidental rotations and resulting torsion introduced during the insertion of real instruments into the silicone phantom. The average distance error between the simulated and scanned instruments was 1.34 ($\pm 0.95$) mm with a root-mean-squared (RMS) error of 1.66 mm. Our method shows nearly a 60% increase in accuracy in comparison with a previous study [25] based on a mass-spring model, when tested on the same real instrument data set. The average RMS error is slightly higher than the 1.25 mm of the Cosserat rod-based guidewire simulation presented in [34].

**Fig. 7:**
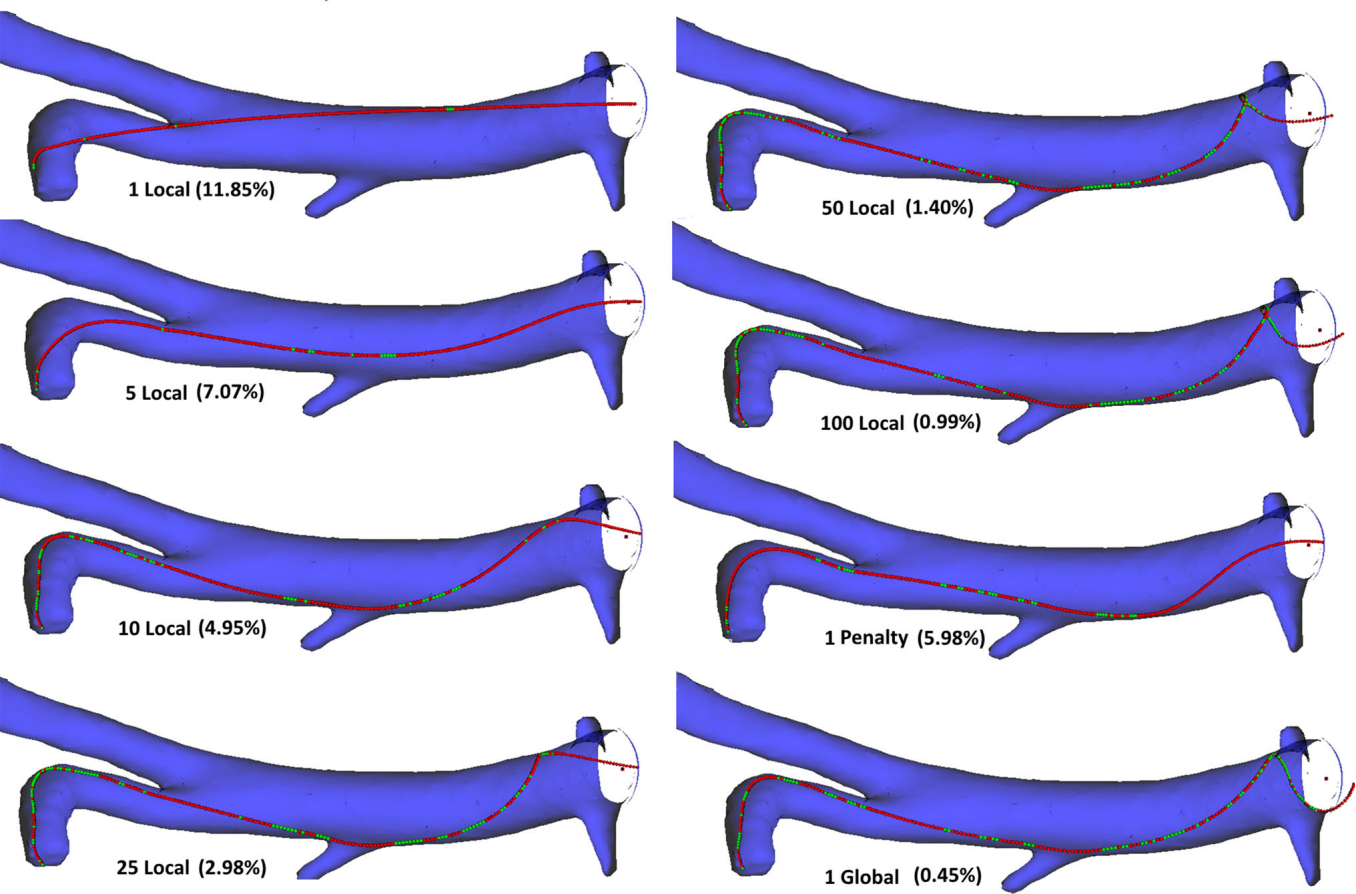
Rod compression with respect to different number and type of distance constraints iterations. The resulting percentage of compression is given in brackets. The green spheres represent colliding mass points and the red ones—non-colliding

To compare the extensibility of our modified model to the original CoRdE model, we gradually inserted a 255-mm rod into a virtual anatomy model. When the rod reaches a bend in the anatomy, its compression behaviour can be measured as it continues to be pushed further in, as shown in Fig. 7. We tested a range of different solvers and number of iterations for our modified model, including local and global Gauss–Seidel methods and a Jacobi approach. The difference between local Gauss–Seidel and local Jacobi is that in the former the updates made by a constraint are immediately visible by a subsequent one. In the latter, the changes are first accumulated in a temporary array and then the actual updates are made. A summary of results of the compression percentages and corresponding computational time for all models are presented in Table 1. The computational time measurements were taken on a HP x4600 workstation (Intel Core2 Quad @2.66 GHz, 8 GB RAM) running Windows $7\times 64$.

**Fig. 8:**
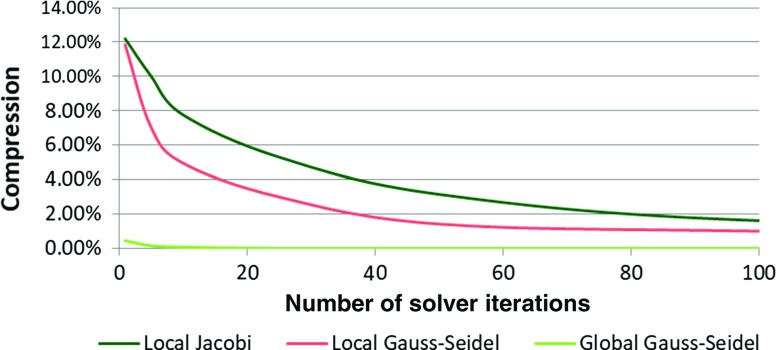
Rod compression in respect to the number of solver iterations

These results showed that using the global block iterative Projected Gauss–Seidel solver, running a single iteration over the constraints, caused the rod to be compressed by 0.16 mm (0.45%). In contrast, the CoRdE model with the largest, stable stretching Young’s modulus, compressed by nearly 14.6 mm (5.98%). Such a compression is not only clearly noticeable, but can also affect rod trajectory. Furthermore, the results demonstrated that a single iteration using the global approach was sufficient to provide a visually inextensible rod at low computational cost (0.052 ms). Figure 8 highlights that increasing the number of solver iterations does not significantly improve the rod simulation quality. Twenty-five iterations fully eliminated the rod compression, yet such a number is not feasible for real-time applications as the computational time is 1.24 ms.

**Table 1:** Rod compression and constraints solver computational times using local and global approaches to distance constraints compared to CoRdE

Distance constraints method	Compression (%)	Time (ms)
Penalty method (CoRdE)	5.98	–
1 Global Gauss–Seidel	0.45	0.052
5 Global Gauss–Seidel	0.15	0.25
10 Global Gauss–Seidel	0.07	0.48
25 Global Gauss–Seidel	0.00	1.24
1 Local Gauss–Seidel	11.85	0.017
10 Local Gauss–Seidel	4.95	0.16
100 Local Gauss–Seidel	0.99	1.555
1 Local Jacobi	12.19	0.021
10 Local Jacobi	7.78	0.16
100 Local Jacobi	1.63	1.62

Figure 8 confirms that both local approaches exhibit an inverse exponential numerical convergence characteristic and, as expected, the Jacobi solver convergence is slower. Compression reduction below 5% using these methods quickly becomes not feasible. Even after 100 iterations, the accuracy of a single iteration using the global approach is still lower. However, the advantage of this approach is that computations can be done in parallel and therefore might be more suitable for a massively parallel GPU implementation.

**Fig. 9:**
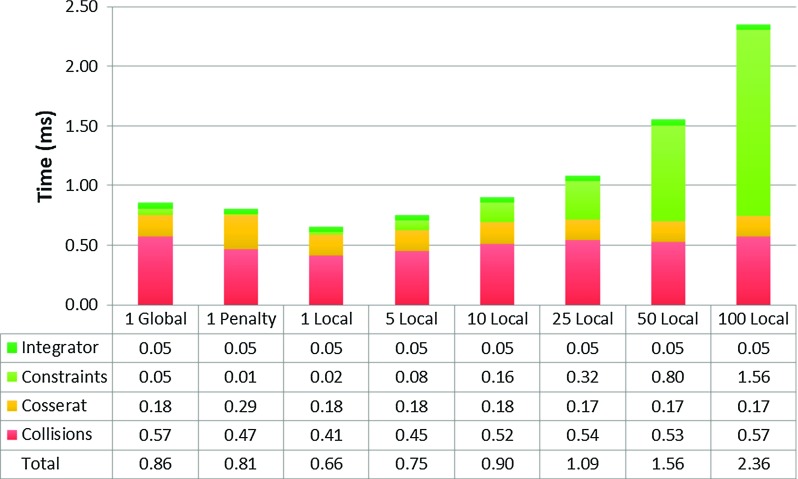
The computational performance of a single rod consisting of 256 mass points

Figure 9 presents a breakdown (numerical integration, constraints calculation, Cosserat rod forces calculation and collision detection) of the total computational time with respect to the solver type and number of distance constraint iterations in the compression test. Self-collisions were not considered.

The total computation time of a single global iteration (0.86 ms) is closest to 10 local iterations (0.90 ms). Even though the global constraints calculations are three times faster (0.05 vs. 0.16 ms), an increased number of collisions caused by the lower rod compression reduces the performance (0.57 vs. 0.52 ms). The local constraints calculation time increases linearly in respect to the number of iterations. For both 1 global and 10 local iterations, the collision detection is a dominant part of the calculations, taking 67 and 58% of the total calculation time, respectively, which causes a serious performance bottleneck. Fortunately, the collision detection may be separated into a different thread (task parallelism).

**Fig. 10:**
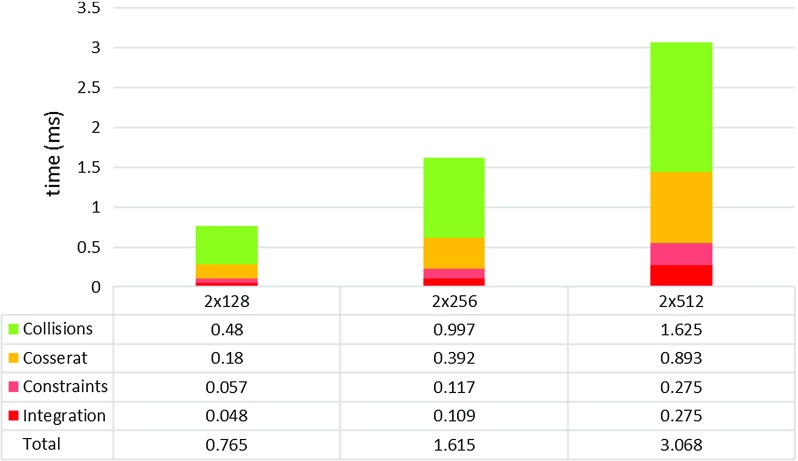
Averaged computational times in ms including instruments interactions achieved during insertion of guidewire/catheter pair into a silicone phantom model for different rod lengths

The experiment was performed a second time to evaluate the behaviour of a guidewire and catheter pair together using a single global GS iteration for constraint solving. The guidewire was inserted into the model first, followed by the catheter. The guidewire was kept inside the catheter using concentric constraints. Figure 10 shows the computational performance of the instrument pair interacting with each other using the same workstation (Intel Core2 Quad @2.66 GHz, 8 GB RAM). Self-collisions were not considered.

The results show that the concentric constraints introduced a minimal performance overhead. Similarly to the previous test, the collision detection took the majority of the computational time. The rods consisting of 512 mass points cannot run at the minimum haptic interactive rates (500 Hz, $<2$ ms per physics step) required for smooth force feedback. However, as mentioned before, collision detection could be allocated to a different thread. For the $2\times 512$ case, this would result in a total physics computation time of 1.44 ms, which would still meet the minimum haptic interactivity requirement.

### Validation

An experimental study was designed to assess the face and content validity of the VCSim3 simulator. Participants were required to complete a cardiovascular intervention five times. Specifically, to navigate the catheter and guidewire from the femoral artery into the heart coronaries, localize the stenosis and deploy a stent. Prior to performing their first procedure, all participants were given a technical instruction sheet outlining the nature of the simulation. The aim was to give a brief overview of the equipment, simulator software, functionality and task to perform. After reading the instruction sheet, participants were given a maximum of 2 minutes to familiarize themselves with the manipulation of instruments. They were given the opportunity to ask questions relating to the practicalities of the simulation, but were not allowed to request any technical advice as to how best to perform the procedure.

#### Inclusion criteria

From discussions with several endovascular clinicians, it is recognized that a clinician in training would need to perform a minimum of 300 procedures as the main operating clinician, either with or without senior supervision, in order to gain proficiency. Given that in the UK trainees perform an average of 20 procedures per week, our inclusion criterion is that subjects must have performed endovascular procedures for at least one year. Face and content validity were evaluated by asking participants to complete a questionnaire after the study. They were asked to respond using a five-point Likert scale from 1 (strongly disagree) to 5 (strongly agree). In addition, they were given the opportunity to provide free text comments. The study was given a favourable ethical opinion for conduct by the Imperial College Research Ethics Committee (ref. ICREC_14_2_9).

17 participants (15 males and 2 females) were recruited for the study. All of them described their medical specialization as “cardiology”. Table 2 summarizes the previous experience of the participants.

**Table 2:** Participants postgraduate year of training and previous experience

	Average	Median	Min	Max
Postgraduate year of training (PGY)	7.12	7	2	13
Procedures in humans	1166.18	600	5	5000
Procedures on VR simulators	6.94	2	0	50

#### Face validation

For face validity, 11 questions related to the behaviour of the virtual instruments and their interactions with the vessels were asked, as given in Table 3.

**Table 3:** Face validity—queries regarding instruments behaviour

Face validity—instruments behaviour	
Q1:	The lengths of catheter/guidewire were realistic par
Q2:	The catheter/guidewire bending behaviour was realistic
Q3:	The catheter/guidewire twisting behaviour was realistic
Q4:	The catheter/guidewire (non-)stretching behaviour was realistic
Q5:	The catheter/guidewire tip behaviour was realistic
Q6:	The catheter/guidewire body (shaft) behaviour was realistic
Q7:	The interactions between catheter and guidewire were realistic
Q8:	The interactions between catheter/guidewire and heart vessels were realistic
Q9:	The delay between physical manipulation and visual reaction was realistic
Q10:	The haptic force feedback felt realistic
Q11:	Overall, the catheter/guidewire behaviour felt realistic

**Fig. 11:**
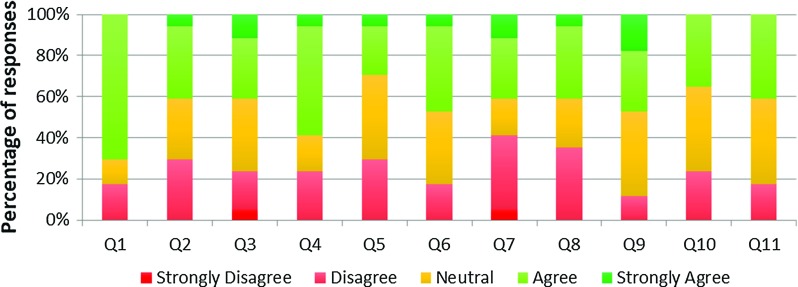
VCSim3 face validity of instruments behaviour—Likert scale responses

Figure 11 shows the results of these questions for all participants. 70% agreed that the lengths of the instruments were realistic (Q1). In terms of questions assessing the physical behaviour of the instruments (Q2–Q6), 43% of responses were “agree” or “strongly agree”, 32% were neutral, 24% “disagree” and 1% “strongly disagree”. In terms of questions assessing interactions between instruments (Q7) and vessels (Q8), 41% of responses were “agree” or “strongly agree”, 21% were neutral, 35% “disagree and 3% “strongly disagree”. Nearly half (47%) of respondents were positive or very positive about the latency (Q9) and 35% were affirmative about the haptic force feedback (Q10). In both statements (Q9 and Q10), 41% of participants were neutral with 12% and 24% disagreeing. 41% of participants agreed that, overall, the catheter/guidewire behaviour was realistic (Q11) with 41% being neutral and 18% disagreeing. In total 45% of the responses to the questions assessing the realism of the behaviour of the instruments (Q1–Q11) were “agree” or “strongly agree”, 30% were “neutral”, 25% “disagree” and 1% “strongly disagree”.

Examples of the free text comments are given in Table 4 and highlight some of the problems that can cause a lack of realism in the instrument’s behaviour.

**Table 4:** Face validity—free text comments

Face validity—free text comments regarding instruments behaviour	
“The overall catheter/guidewire interaction was quite realistic. However, the manipulation of the catheters was not so realistic and they didn’t feel like real catheters as they moved too smoothly”	
“The guidewire torque was not realistic as it was very heavy compared to normal”	
“The wire popped out of the catheter unexpectedly on a frequent basis in a way it wouldn’t normally do. The appearance of the wire was also not accurate as it appeared to be beaded”	
“Manipulation of the catheters was easier than in real life”	
“The latency was probably a little too short (i.e. too responsive) and the catheters felt a little “floppy”	
“When tried to withdraw the catheter back while fixing the guide wire, the guide wire moved distally in the coronary vessel”	
“Great concept, but still not quite realistic feeling”	

Face validity was also considered in terms of the realism of the supporting solutions. Eight questions were asked regarding this (Table 5). The specific questions were:

**Fig. 12:**
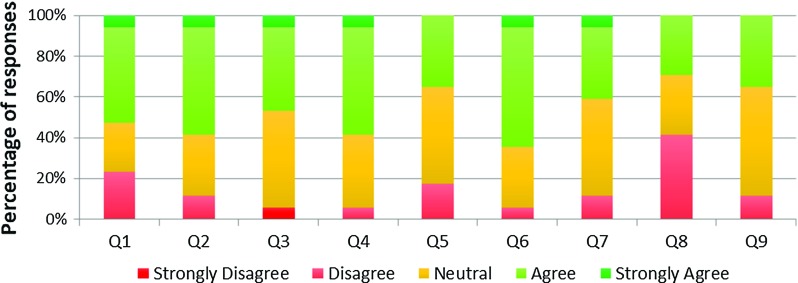
VCSim3 face validity of supporting solutions—Likert scale responses

**Table 5:** Face validity—queries regarding supporting solutions

Face validity—supporting solutions	
Q1:	The contrast medium injection felt realistic
Q2:	The balloon inflation felt realistic
Q3:	The contrast medium propagation was visually realistic
Q4:	The balloon inflation was visually realistic
Q5:	The stent deployment was visually realistic
Q6:	The cardiac motion was visually realistic
Q7:	The visualization as a whole looked realistic
Q8:	The difficulty of the simulated procedure was realistic
Q9:	Overall the simulator was realistic

The results for these questions are presented in Fig. 12 and show that 56% of answers to statements regarding the feel of the contrast medium injection and balloon inflations (Q1–Q2) were “agree” and “strongly agree”, 26% were “neutral” and the remaining 18% were “disagree”. 50% of responses evaluating the visual aspects of the simulator (Q3–Q7) were “agree” and “strongly agree” and 41% were “neutral”. 41% of participants disagreed that the difficulty of the simulated procedure was realistic (Q8), 29.5% were “neutral” and 29.5% “agreed”. 35% of participants agreed that, overall, the simulator was realistic (Q9) with 53% being neutral and 12% disagreeing with this statement.

In total, 47% of all the responses to questions (Q1–Q9) assessing the realism of the supporting solutions were “agree” or “strongly agree”, 38% were “neutral” and 14% “disagree”. Only one response was “strongly disagree”. Table 6 highlights some of the free text feedback received and demonstrate the variety of opinions.

**Table 6:** Face validity—free text responses regarding supporting solutions

Face validity—free text responses regarding supporting solutions	
“It’s a little too responsive and easy. This is true of all the simulators I’ve used. The visualizations were, if anything, too good (i.e. the definition was sharp and the anatomy too easily seen). In the cath lab, we can’t see the vessels we’re aiming for until we find them”	
“Manipulating the guidewire was too difficult. The balloon manipulation and inflation was very realistic”	
“Needs improved tracking/force feedback equipment. The behaviour of the tip of the catheter was very impressive—jumping up, storing torque etc. into the coronary arteries. Also good was how it would occasionally jump up the carotids mirroring cath lab problems. Liked the push back on the catheter that you got when you post the intra-coronary wire down the coronary artery with it sometimes tangling up the shape of the catheter and pulling out the wire—also mirrored the tricks you do to restore the correct positioning”	

#### Content validation

**Fig. 13:**
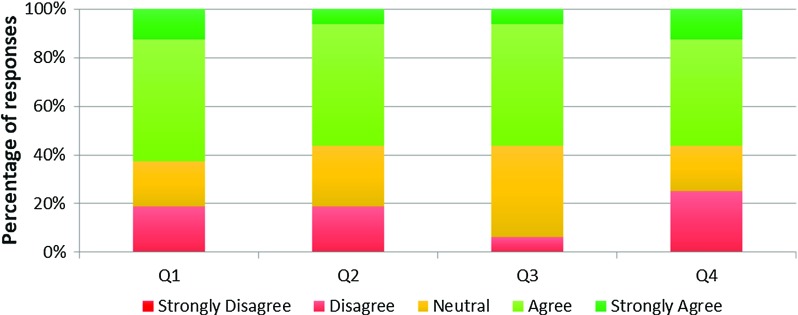
VCSim3 content validity—Likert scale responses

Content validity was also evaluated. The questions asked assessed the adequacy of the simulated tasks and perceived utility of the simulator as a training tool for cardiovascular interventions. Specifically, it contained four questions (Fig. 13).

In total, 58% of the responses to the questions assessing the content validity were “agree” or “strongly agree”, 25% were “neutral” and 17% “disagree”. None of the participants “strongly disagreed” with any of the above statements. Table 7 details the free text comments given regarding the overall content validity and possible steps for improvements (Table 8).

The above results suggest that the VCSim3 demonstrates early signs of face validity in terms of the realism of the instruments. However, given the free text comments from participants, it is likely that the drawbacks of the VSP haptic device affected their perception of the realism of the virtual instruments. The limited catheter tracking length (30cm) is sufficient to navigate the catheter between the aorta and the coronaries; however, inserting the catheter too far can result in a loss of tracking of the guidewire. This causes the guidewire to be unresponsive and requires both instruments to be withdrawn and reinserted, leading to frustration and confusion for the participants. Moreover, the force feedback mechanism inside the VSP is built around a motor which simply clamps the instruments. Such a mechanism does not separate linear force feedback from rotational force feedback. These disadvantages can be potentially solved by using an improved haptic device.

The results of the face validity of supporting solutions, such as fluoroscopic visualization, contrast propagation, cardiac motion, balloon inflation and stent deployment, range mainly from neutral to positive. This suggests that, although technically simple, they provide sufficient functionality and realism in the view of the majority of the participants. In statements relating to content validity, more than 50% of responses were positive or very positive indicating that VCSim3 has the potential to become a useful training tool for cardiovascular interventions. Most of the participants would also recommend it to their colleagues.

**Table 7:** Content validation queries

Content validity	
Q1:	The catheter/guidewire behaviour is sufficient to make it a useful training tool for cardiovascular interventions.
Q2:	The remaining functionality provided by the simulator is sufficient to make it a useful training tool for cardiovascular interventions.
Q3:	Overall the simulator is a useful training tool for cardiovascular interventions
Q4:	I would recommend the simulator to others

**Table 8:** Content validity—free text comments

Content validation—free text comments	
“At a very early stage in training there is probably a role for this in demonstrating the coronary anatomy in relation to different radiological views. Basic intubation concepts and procedural concepts could also be usefully practised”	
“Tactile feedback needs more work”	
“This has huge potential, but trainees could not yet rely on this simulator as a reflection of what happens in patients”	
“So far the simulator is not very realistic and needs a lot of work still. The manipulation of wires is the main problem”	
“Clearly has great potential and the core behaviours are done very well. Needs a few tweaks to the physical interface, and reflect some of the constraints you have in the cath lab—only a few fixed views, less clear images etc”	

## Conclusions

This paper introduces VCSim3—a VR simulator for cardiovascular interventions. A modified version of the CoRdE model is presented to create realistic behaviour of a virtual catheter and guidewire. This allows for efficient modelling of bending, stretching and twisting phenomena, as well as guaranteeing almost immediate response to user manipulations, even for long instruments. The mechanical parameters of six guidewires and three catheters were optimized with respect to their real counterparts scanned in a silicone phantom using CT. The implementation allows the simulator to run efficiently on an off-the-shelf PC or laptop, significantly exceeding the minimum required haptic interactive rate. Results show the parameter-optimized virtual instruments exhibit near-sub-millimetre accuracy, with errors likely to be caused by the accidental rotations and resulting torsion introduced during the insertion of real instruments into the silicone phantom. The global distance constraints used result in a practically inextensible and incompressible rod, as desired.

An initial verification of the simulator was carried out by obtaining subjective feedback (face and content validity) from 17 cardiologists. The results of the face validity suggest that VCSim3 demonstrates early signs in terms of the realism of the simulated instruments. Nearly half of the participants was positive about the behaviour of virtual catheters and guidewires. The results of the face validity of supporting solutions such as fluoroscopic visualization, cardiac motion, contrast propagation, balloon inflation and stent deployment ranged from neutral to positive. In terms of content validity, more than half of responses were positive or very positive indicating that the majority of the participants agreed that VCSim3 is a useful training tool for endovascular interventions, and that they would recommend it to others.

In terms of limitations, VCSim3 suffered from the restrictions of the commercial haptic device. The number of available procedures is also currently limited to angioplasty and stenting of left and right coronary arteries extracted from a single patient-specific CT dataset. The catheter and guidewire behaviour, although on average positively acknowledged by the participants, received more mixed feedback than the virtual endoscope in NOViSE [39]. This could be due to the subtler nature of the guidewires and catheter manipulation, as well as the shortcomings of the haptic device. Therefore, more investigation is needed to identify subtle phenomena occurring during the real endovascular procedures.

The supporting solutions implemented in the simulator, although visually plausible and positively rated by the participants, lag behind the state of the art. Our future work plans include improvements to X-ray visualization by approximating physical phenomena, which takes place during CT scan as well as streamlining the vessels segmentation with support of cardiac motion. We are also investigating methods of deformation of the coronaries, which can straighten when stiffer guidewires are inserted inside them. However, supporting both motion and deformation of the coronaries in a realistic and real-time manner remains challenging.

The face and content validation study suffered from relatively small number of participants and the construct validity was not assessed. Therefore, we intend to conduct a further study with a larger number of participants that also includes construct validation, after addressing the identified shortcomings.

## Electronic supplementary material

Below is the link to the electronic supplementary material.

Supplementary material 1 (mp4 314835 KB)

Supplementary material 2 (mp4 1856 KB)

Supplementary material 3 (mp4 1375 KB)

Supplementary material 4 (docx 3953 KB)
