# Supplementary material for: VCSim3: a VR simulator for cardiovascular interventions
Source: Int J Comput Assist Radiol Surg. 2017 Oct 27;13(1):135–49. doi: 10.1007/s11548-017-1679-1 (PMC5754385; doi:10.1007/s11548-017-1679-1)

Validation of VCSim 3.0

A virtual reality simulator for cardiovascular interventions

Research Protocol

Researcher name: Mr. Przemyslaw Korzeniowski

Supervisor: Dr Fernando Bello
Reader in Surgical Graphics & Computing
Division of Surgery, Dept. of Surgery and Cancer

Imperial College London

February 2014

# Introduction

Cardiovascular diseases or CVD are the number one cause of death around the world [1]. It is estimated that 17.3 million people died from CVDs in 2008. The key advent of pinhole surgery at the end of the 20^th^ century has enhanced the diagnosis and treatment of many major vascular diseases and has become a vital part of vascular health care today. With this approach, patients suffer much less tissue trauma, which leads to faster recovery and reduced treatment costs since they can usually be treated as day surgery cases [2]. Endovascular clinicians require extensive training and practise because endovascular procedures demand dexterity in handling the delicate guide wire and catheter tools and good hand-eye coordination.

Endovascular clinicians are largely trained using the traditional apprenticeship model where the trainee or apprentice learns first through observation, and then by gradually assisting and performing surgical procedures themselves under the direct supervision of the senior clinician. Several factors such as the increasing costs of time in the operating room [3] have resulted in a need for alternative out-of-the OR training methods such as virtual reality (VR) simulators [4]. Virtual reality simulation has been shown to be a safe and effective training. These simulators have the advantage of being adaptable to simulate different anatomies, as well as having haptic feedback that helps to recreate the feeling of handling the tools through the sense of touch. We have therefore developed a virtual reality cardiovascular simulator - *VCSim*, which allows operators to develop skills in a safe environment and provides them with objective feedback on their performance.

The aim of this study is to establish face, content and construct validity for our simulator in performing cardiovascular interventions. We intend to achieve this by conducting a study that involves gathering the opinions from medical practitioners through an online questionnaire and an experimental study using a prototype of the simulator. The only ethical considerations for the study are voluntary participation, data confidentiality, anonymity and use of the gathered data. This study does not involve patients. It requires only the consensual participation of medical practitioners. The identities of the participants will be anonymised and their responses and any other gathered data will be kept confidential at all times in accordance with the Data Protection Act.

## References

[1] Global status report on noncommunicable diseases 2010. Geneva, World Health Organization, 2011.

[2] Benefits of Minimally Invasive Surgery, University of Chicago Webpage, accessed 17 Oct 2013. http://www.uchospitals.edu/specialties/minisurgery/benefits/

[3] The financial impact of teaching surgical residents in the operating room, Bridges, M. ,Diamond, D.L., American Journal of Surgery, 1999 Jan, 177(1), 28-32.

[4] Simulation in Surgical Education, S. de Montbrun, H.MacRae. Clin Colon Rectal Surg,25(3), September 2012, 156-165.

# Study Aim & Objectives

The aim of this study is to establish face, content and construct validity for our simulator in performing cardiovascular interventions. The main focus will be put on the behaviour of virtual instruments – catheters and guidewires. Specifically, we want to investigate if the instruments stretch, bend, twist and interact with heart vessels in a realistic way, appropriate for training.

Additionally, we want to examine other aspects of the simulator such as:

- Visual output
- Contrast flow propagation
- Cardiac motion
- Balloon inflation
- Stent deployment
- Haptic feedback

# Study Design

We aim to recruit 20-30 participants meeting the entry criteria. Participants’ operative experience, demographic data, interest in virtual reality simulation and experience of videogames will be recorded with an online questionnaire.

The experimental apparatus consists of a physical, force feedback human-computer-interface (the haptic device) and a real-time software simulation (the simulation). The complete experimental set-up is presented in Fig. 1 and Fig. 2 in the Appendix.

The simulation software during each update step:

- receives operators movements from the haptic device
- calculates the motion of the virtual catheter and guidewire
- processes the interactions of the catheter and guidewire with the vessels
- measures and stores the performance metrics
- calculates and sends the force feedback back to the haptic device

The software constantly tracks and stores all the movements of the haptic device and of the virtual instruments. The software also stores the metrics such as procedure completion time and applied forces.

## Simulation

All participants will be required to complete 5 cardiovascular interventions. Specifically, to navigate the catheter and guidewire from the femoral artery into the heart coronaries, localize the stenosis and deploy a stent. The participants’ performance will be analyzed in relation to all their operative attempts and an average will be taken. Prior to performing their first procedure, all participants will be given a technical instruction sheet (*Appendix F*) outlining the nature of the simulation. The aim of this sheet is to give a brief overview of the equipment, tasks and factors, which will differ to real life owing to the limitations of performing the procedure in a simulated setting. After reading the instruction sheet, participants will be given a maximum of 2 minutes to familiarize themselves how to operate the instruments. Prior to commencing their first recorded procedure, participants will be given the opportunity to ask questions relating to the practicalities of the simulation, but will not be allowed to request any technical advice as to how best to perform the procedure. No time limit will be set for the recorded procedures.

## Participant data

Participants’ operative experience, demographic data, interest in virtual reality simulation and experience of videogames will be recorded with an online questionnaire (<https://www.surveymonkey.net/s/vcsim>, *printed copy in Appendix A*).In addition, we may also video record the procedure (computer screen and instruments manipulation). If participant wish to withdraw from the study all data related to their participation will be permanently deleted.

## Face validity

Face validity will be evaluated by asking participants to complete the aforementioned questionnaire after completing all procedures. The questionnaire will assess the behavior of instruments, graphical appearance, difficulty of the procedure and overall realism.

## Content validity

Content validity will be evaluated by asking participants to complete the aforementioned questionnaire after completing all procedures. The questionnaire will assess the adequacy of the simulated tasks and perceived utility of the simulator as a training tool for cardiovascular interventions.

## Construct validity

Construct validity will be evaluated by comparing operative performance metrics of participants.

## Intended outcome

It is hoped that the study will lead to the face, content and construct validation of the *VCSim* simulator prototype.

## Participant Entry Requirement

### Inclusion Criteria

The subjects in this study are also known as endovascular clinicians. They are medical professionals that have been trained or are still in training as interventional radiologists, vascular surgeons and interventional cardiologists. From discussions with several endovascular clinicians, it is recognised that a clinician in training would need to perform a minimum of 300 procedures as the main operating clinician, either with or without senior supervision, in order to gain proficiency. Given that in the UK trainees perform an average of 20 procedures per week, our inclusion criteria is that subjects must have performed endovascular procedures for at least one year.

### Exclusion Criteria

Subject has not performed endovascular procedures for a minimum of one year.

### Withdrawal Criteria

This is a non-intervention study and there are no consequences for early withdrawal. The subject or participant may withdraw consent at any point.

## Data Management

### Simulation

Each participant will be assigned a unique identification code. Their data will be carefully anonymised to remove all personal identifiers except for their professional background, position and years of experience. This data will be stored on a secure Imperial College server. There will be a journal/notebook linking this unique code with the individual in case there is a need to go back and look at the personal details again or to exclude a participant’s data. The journals will be stored in a locked filing cabinet in a secure office by the data custodian. The appointed data custodian will be Dr Fernando Bello (the study Chief Investigator).

### Online questionnaire

The online questionnaire does not require the participant to provide any details that reveal their personal identity other than their medical background and years of professional experience. It is therefore not possible to link the responses of the questionnaire to a specific participant. This ensures the anonymity of participants in the study. Questionnaire responses will be stored online in a secure online account ([www.surveymonkey.com](http://www.surveymonkey.com)).

## Adverse Events

This study is not a clinical trial of an investigational medicinal product or medical device. It involves no drugs and no novel procedures. It involves qualitative observations and recordings. Therefore no adverse events are expected.

## Assessment and Follow Up

There will be no clinical intervention and therefore no follow-up intervention required.

## Data Analysis

To ensure the external validity of the study, we aim to recruit a minimum of 20 participants to complete the online questionnaire. All data will remain anonymous. Data will be analyzed using a statistics package (Statistical Package for the Social Sciences (SPSS) version 18.0.1). Descriptive statistics and frequencies will be calculated with appropriate methods according to the type of data. Significance will be set at p<0.05.

# Regulatory Issues

## Ethics

The main ethical considerations refer to voluntary participation, data confidentiality, anonymity and use of the gathered data. To address participation, an information sheet will be provided to ensure participants are adequately informed. The participants will be assured that the data collected will be confidential, remain anonymous and be used solely for the purposes of this study. The collection and handling of the data will be in accordance to the Data Protection Act.

The research will not involve work done under the Animals (Scientific Procedures) 1986 Act. The research will not involve the use of genetically modified tissue. The project will not involve the use of post-operative, post mortem material or access to confidential patient information.

This protocol will be submitted to the ICREC for ethical approval because this work does not involve NHS patients.

## Consent

All participants will be required to sign a written consent form prior to the commencement of the study and will be free to withdraw from the study at any point.

## Funding

The development of the VCSim prototype was funded by the EPSRC. Current testing and validation is funded through Health Education England and ICHT.

## Study Management

The day-to-day management of the study will be co-ordinated by the researcher Mr Przemyslaw Korzeniowski under the supervision of the Chief Investigator, Dr Fernando Bello.

## Publication Policy

Only anonymised data will be used in publication. It is anticipated that resources acquired through the study may be included in presentations at conferences and publications in peer-reviewed journals.

# Appendix

A. Online Questionnaire Form

B. Information Sheet for Participants

C. Consent Form for Participants

D. Recruitment Email for Participants

F. Technical Instruction Sheet for Participants


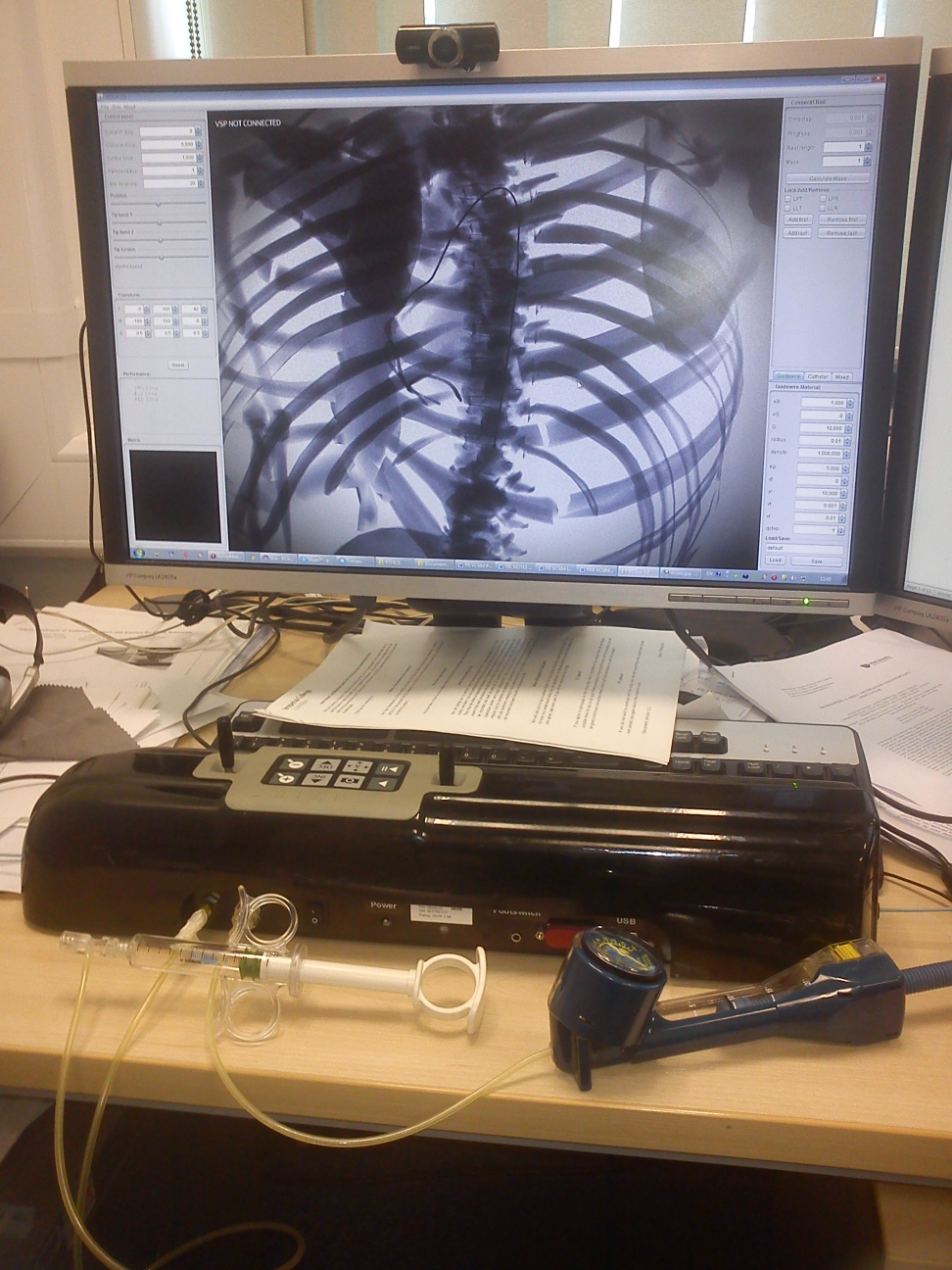


Figure 1: A complete experimental set-up. On the desk, the haptic device (black box) with a syringe and a balloon inflation device connected. On the computer screen, a running simulation software.


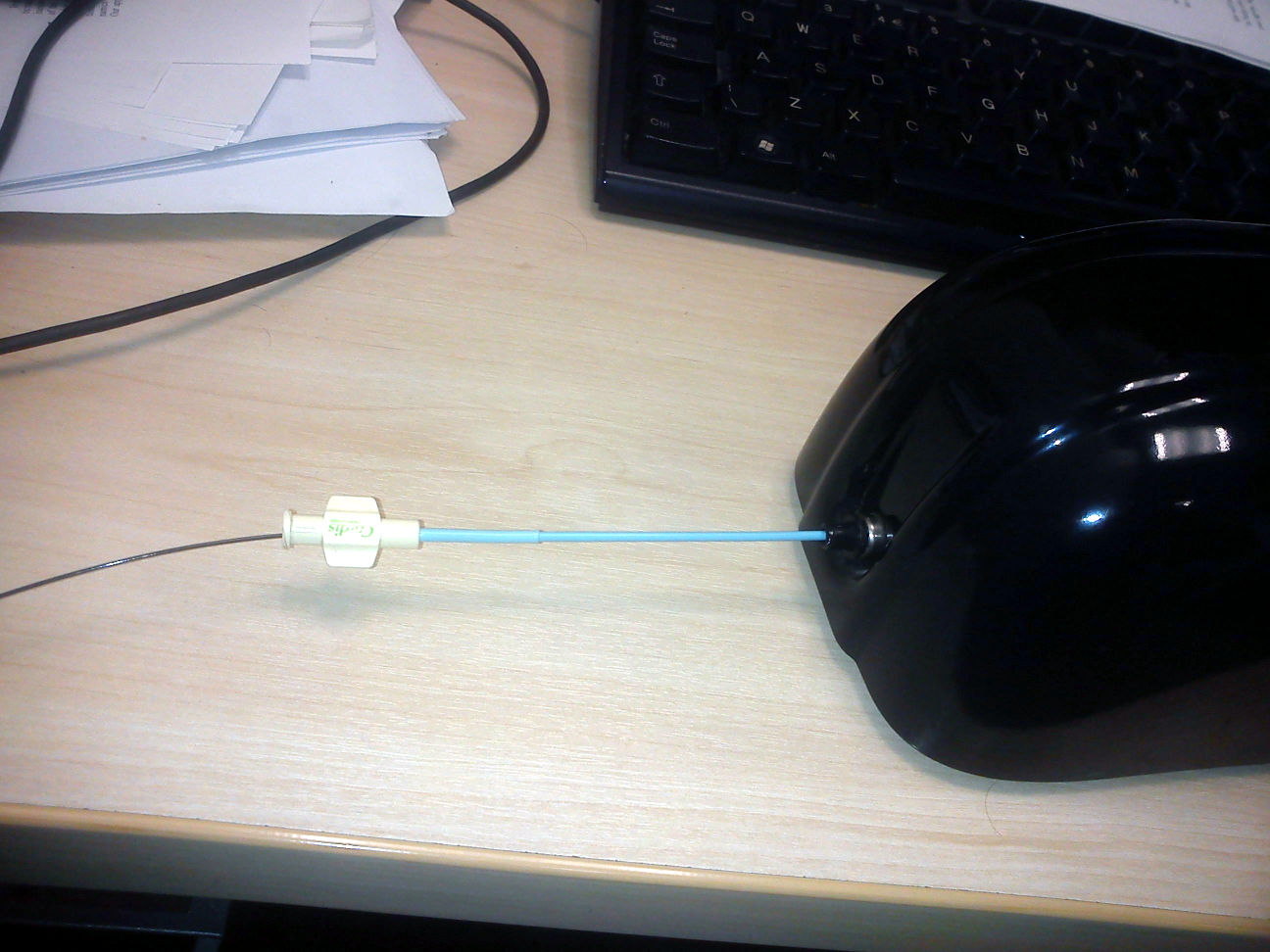


Figure 2: A zoom in at the real instruments (a guidewire inside a catheter) inserted into the haptic device (VSP).

# Appendix A – Online Questionnaire Form


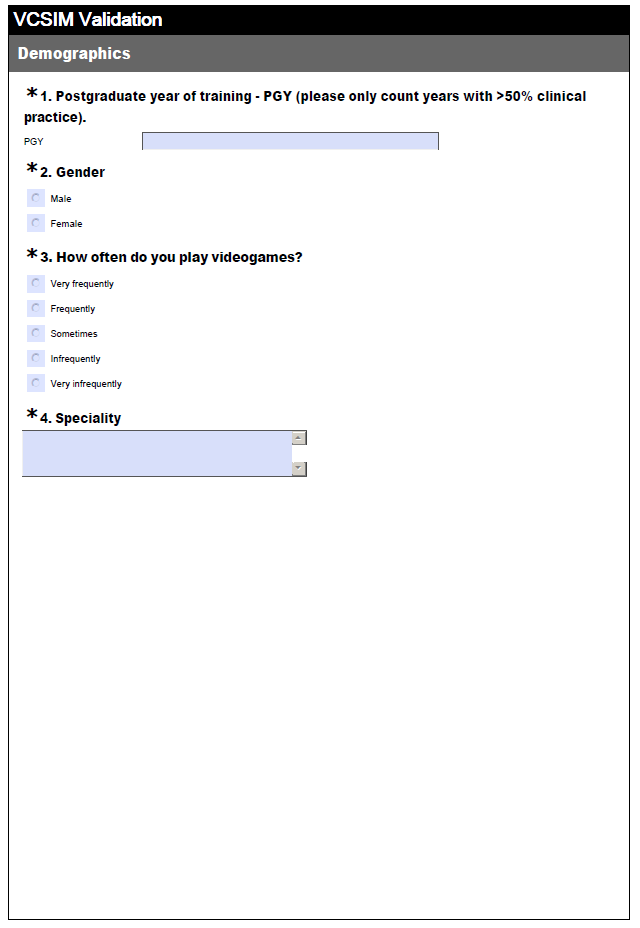

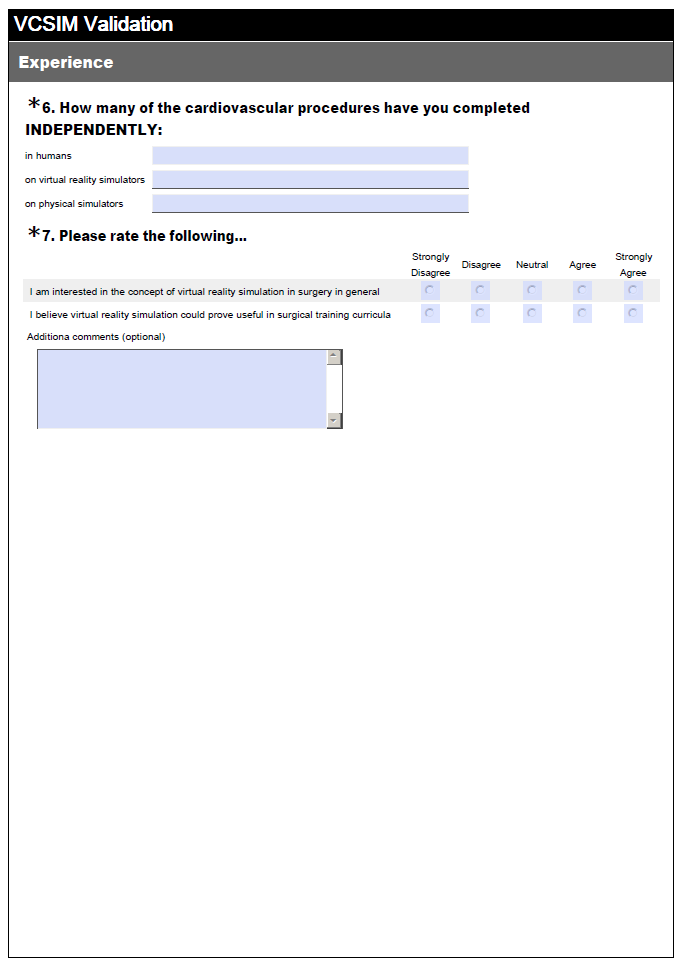


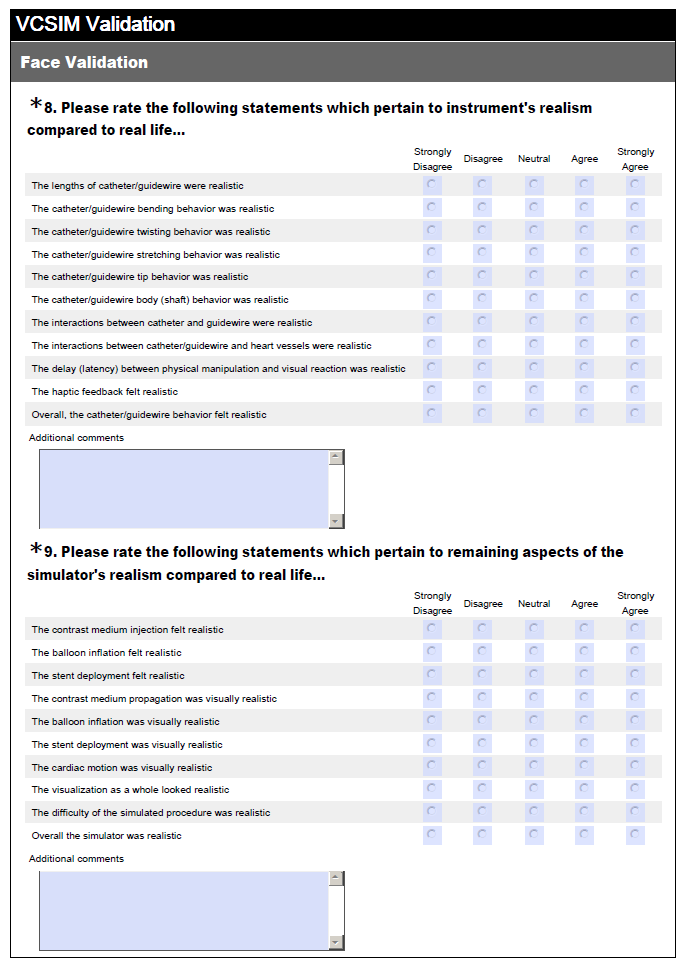


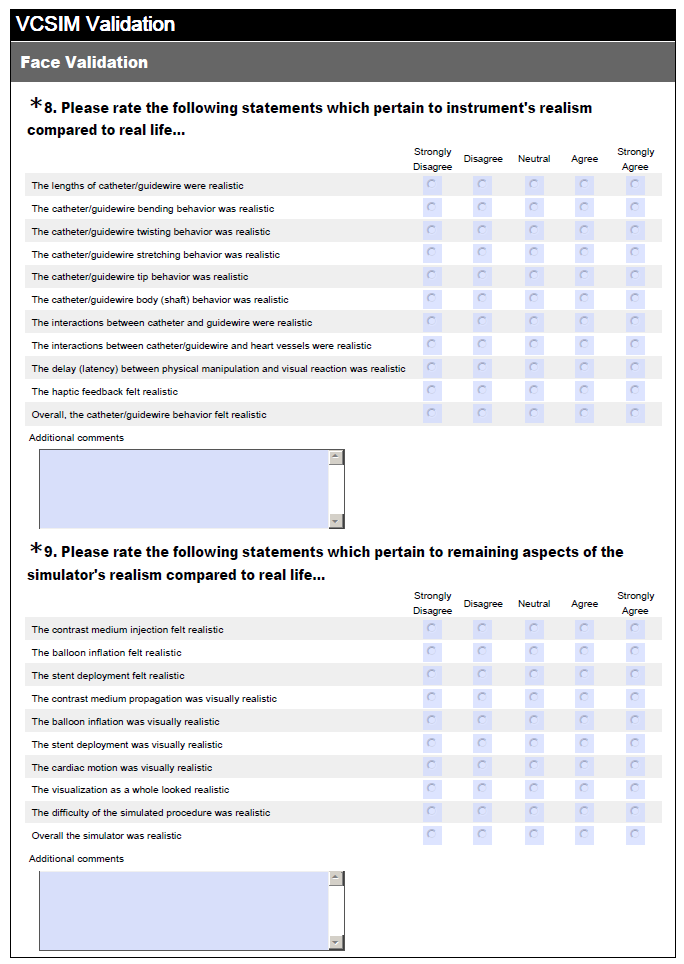

Supplement: Supplementary file 4 — Supplementary material 4 (docx 3953 KB) [file 11548_2017_1679_MOESM4_ESM.docx]
